# Literary Gossip and Record

**Published:** 1862-04

**Authors:** 


### Art. X.—LITERARY GOSSIP AND RECORD.

Another name must be added to the long list of those medical worthies who have won fame with the pen in other than professional fields of literature. *Notes on the Chase of the Wild Red Deer in the Counties of Devon and Somerset, with an Appendix on Remarkable Runs and Incidents connected with the Chase, from the year* 1780 *to the year* 1860, by Charles Palk Collyns, of Dulverton, Surgeon, is one of those rare books which, while charming the present generation of readers, will have a permanent historical value as a trustworthy record of the red deer in probably the last of its native haunts in the South of England. We can imagine sportsmen, naturalists, and historians of social manners, in generations yet to come, poring over Mr. Collyns' pages with the same delight with which he pores over the quaint descriptions of Master John Manwood, in his "Treatise on the Laws of the Forest."

The wild stag still runs over the high and desolate moorland region of Exmoor, and the open lands stretching away to the Quantocks, a district flanking the Bristol Channel, and extending from north to south forty miles, and east to west above fifty miles. In the ancient royal forest of Exmoor the preservation of the red deer was an object of solicitude to William the Conqueror, who, the Anglo-Saxon Chronicle tells us, "Loved the tall stags as if he were their father" (a character which appears to have escaped Mr. Collyns' notice). The association of the Conqueror's name with the chase and forest laws is not, however, a pleasant one, as it brings too forcibly to mind the barbarous circumstances which attended the origin of those laws in England, and which cast a lurid light over their subsequent history throughout several centuries. "He" (the Conqueror), states the Anglo-Saxon Chronicle, "made large forests for the deer, and enacted laws therewith, so that whoever killed a hart or a hind, should be blinded." In what manner these forests were formed, Holinshed thus tells us, in one instance:—"He pulled down townes, villages, churches, and other buildings for the space of thirty miles, to make thereof a forrest, which at this daie is called New Forrest. The people then sore bewailed their distress, and greatly lamented that they must thus leave house and home to the use of savage beasts. Which crueltie, not onlie mortall men living here on earth, but also the earth might seeme to detest as by a wonderful signification it seemed to declare by the shaking and roaring of the same which chanced about the fourteen yeere of his reign (as writers have recorded)." The Anglo-Saxon Chronicle says,—"The rich complained and the poor murmured; but he was so sturdy that he recked nought of them; they must will all that the king willed, if they would live, or would keep their lands; or would hold their possessions; or would be maintained in their rights. Alas! (adds the pious chronicler) that man should so exalt himself and carry himself in his pride over all! May Almighty God show mercy to his soul, and grant him the forgiveness of his sins! We have written concerning him these things both good and bad, that virtuous men might follow after the good, and wholly avoid the evil, and might go in the way that leadeth to the kingdom of heaven."

Now, it is to be remembered, that when the "depopulations" (as the older writers forcibly term the Conqueror's method of preparing "chases" for his pastime) occurred, famines were frequent and "cruel" (Oh, how disastrous, how rueful were those times!"), and the crops of one season at the best would rarely suffice to do more than furnish the requirements necessary for existing until the season following. Hence the Conqueror's savage enactment concerning the killing of deer, and which applied also to other beasts of the chase, while even hares were defended from harm, is to be looked upon, on the one hand, as a measure of the incentives required to keep a famishing population from satisfying its cravings for a readily accessible food, and, on the other, as a means of preventing their taking summary measures for preventing depredations upon their crops. Mr. Collyns furnishes a significant illustration of the destructiveness of the deer in this respect. "The damage done by deer," he says, "in feeding in inclosed and cultivated lands, is very great. They destroy more than they consume; and I have seen five or six cartloads of turnips pulled up in a single field by the marauders, in the course of a morning's meal. How much are the sportsmen of the west," he adds, "indebted to the kind and unselfish feelings of those farmers who endure this loss and annoyance, in order that they may contribute to the amusement of their friends and neighbourhood."

But the period is rapidly coming when the red deer will vanish altogether from its last accustomed haunts in the South of England.

"There was a time," says Mr. Collyns, "when the hills and vales of South Devon echoed the notes of the stag-hunter's horn, and history could tell how many a good stag, found on the southern slope of the Dartmoor Hills, gave up his life in Torbay, or took soil in the rippling streams of the Dart. But the progress of civilization, increase of population, and modern improvements in farming, with the consequent inclosure and cultivation of tracts of land once waste and quiet, have combined in effecting the extinction of deer; and it may be safely asserted that there is not a single specimen to be found at the present moment either in the south of Devon or Cornwall. That they existed in the last-mentioned county, until a comparatively recent period, there can be no doubt, although their extermination is now effectually completed. Such would not have been the case if the taste, which formed one prominent feature in Dutch William's character, had animated his royal successors. We read that this monarch imposed a fine of six hundred pounds on Lady Athlone for having killed six stags, at different times, near Loo, and salted them down for her servants' use. As the red man retreats before his pale-faced brethren to wilder prairies and more secure fastnesses, so the deer have sought, as their last abiding-place, the wastes of Exmoor and the purlieus of the forest. Within fifty years the barren tracts and woods around Bagshot sheltered the wild red deer; but there they have ceased to exist. Until recently they were preserved in the New Forest; but the order for their extermination there has gone forth, and is, I believe, executed, and there is probably no place in England, at least south of the Humber, in which a wild stag can be seen, save in the north of Devon and western part of Somerset. The space over which they can roam undisturbed in the west is, however, becoming more and more limited—the ploughshare creaks where, but a few years ago, the bittern boomed, the heron screamed, and the plover whistled in the sedgy morass—the long-drawn furrow and the busy measuring-chain predict further enclosures and renewed encroachment on the habitation of the dun deer; and I fear that I am a true prophet in foretelling that there are sportsmen now alive, who, in their old age, will tell of stag-hunting as of a thing that was—as a sport which they remember, but which has passed away. If my feeble efforts to excite an interest in the minds of those who are able to retard the extermination of the animal, by a sketch of the history of the sport, can prolong the existence of the herd for even a few years, I shall think that I have not written in vain; and I shall, also, feel it matter of congratulation, if the perusal of these pages should have the effect of inducing any real lover of sport to witness a stag-chase over Exmoor while he may. I should be selfish, indeed, did I not desire that an amusement, which has interested me beyond all others since my childhood, should be participated in by all who love the chase, whose hearts thrill at the sound of a 'challenge,' and whose pulses beat quicker as they take their horses by the head to strive for superiority over the open."

Mr. Collyns writes in a green old age. More than three-score years have passed over his head, and his life has been spent in the arduous duties of his profession. For forty-six years he has hunted with the stag-hounds, and he now gives to the world, with commendable enthusiasm, the results of his long experience in the chase of the wild red deer, and his large knowledge of that animal—"the most statliest beast in his gate, that doth go upon the earth," as Master Manwood says, "for he doth carry a majesty in his countenance and gate." But Mr. Collyns looks at the noble beast only through the medium of the chase. How its majestic front may not save it from the worry of the hounds, or its supplicating eye from the misery of the chase, being no sportsman, we cannot explain. "To my mind," Mr. Collyns remarks, "the eye of the deer is unequalled by that of any other animal with which I am acquainted. Not even the eye of the gazelle is, in my opinion, so beautiful as the full, dark, thoughtful orb of the deer. 'It displays a peculiar weeping aspect, more observable in the stag than in the hind.'" (P. 60.) We know few things so painful as the look of an animal dying from injury. We have seen tried soldiers unmanned, and been unmanned ourselves, amidst all the excitement of the carnage, by the aspect of wounded horses on a battle field, and we should not like to expose ourselves to a sight such as this:—the stag has taken the water, a boat is manned, a rope thrown round the antlers, the animal dragged "in triumph to the beach, the knife is at his throat, and amid the baying of the pack, and the loud whoo-whoops of the crowd, the noble and gallant animal yields up his life." (P. 145.) We would rather, indeed, for our own part, indulge in the melancholy conceits of Jacques, or in the curious, hyperbolic fancies of Waller, when contemplating the frontlet of a stag:—

"So we some antique hero's strength
Learn by his lance's weight and length;
As these vast beams express the beast,
Whose shady brow alive they drest.
Such game, while yet the world was new,
The mighty Nimrod did pursue.
What huntsman of our feeble race,
Or dogs, dare such a monster chase;
Resembling, with each blow he strikes,
The charge of a whole troop of pikes.
O fertile head! which every year
Could such a crop of wonder bear!
The teeming earth did never bring
So soon, so hard, so huge a thing;
Which might it never have been cast,
(Each year's growth added to the last),
These lofty branches had supply'd
The Earth's bold sons' prodigious pride;
Heaven with these engines had been scal'd,
When mountains heap'd on mountains fail'd."

Mr. Collyns' work will not probably bring about the result which obviously he has most at heart, the preservation of the deer on Exmoor; but he will have the consolation to think, that it will be read, even by those who, like ourselves, have no "sporting" predilections, as a charming piece of contemporary social history, and that it will prove valuable as a record of the last haunt and last herd of wild red deer in the South of England. Books like these constitute the salt of historians.

